# Development of mental health first-aid guidelines for depression: a Delphi expert consensus study in Argentina and Chile

**DOI:** 10.1186/s12888-023-04661-8

**Published:** 2023-03-14

**Authors:** Esteban Encina-Zúñiga, Martín Agrest, Thamara Tapia-Munoz, Isidora Vidal-Zamora, Sara Ardila-Gómez, Rubén Alvarado, Eduardo A. Leiderman, Nicola Reavley

**Affiliations:** 1 School of Public Health, Faculty of Medicine, Universidad de Chile, Santiago, Chile; 2 Department of Psychology, Faculty of Social Sciences, Universidad de Chile, Santiago, Chile; 3 Proyecto Suma, Güemes 4130 (1425), Ciudad Autónoma de Buenos Aires, Argentina; 4 Department of Behavioural Science and Health, University College London, London, UK; 5 Facultad de Psicología, Instituto de Investigaciones, Universidad de Buenos Aires, Buenos Aires, Argentina; 6 Consejo Nacional de Investigaciones Científicas Y Técnicas (CONICET), Buenos Aires, Argentina; 7 Department of Public Health, School of Medicine, Faculty of Medicine, Universidad de Valparaíso, Valparaíso, Chile; 8 Departamento de Neurociencias, Facultad de Ciencias Sociales, Universidad de Palermo, Buenos Aires, Argentina; 9 Centre for Mental Health, Melbourne School of Population and Global Health, University of Melbourne, Melbourne, Victoria Australia

**Keywords:** Depression, Mental health first aid (MHFA), Cultural adaptation, Delphi study, Chile, Argentina

## Abstract

**Background:**

Depression is one of the most common mental health problems worldwide and, while prevalence rates in Latin America are relatively high, most people who meet the criteria for diagnosis do not receive treatment. Family and friends of a person with depression can play an important role in supporting a person to seek and engage with treatment. However, many people do not have the necessary skills or confidence to help. English-language mental health first aid guidelines have been developed to support people to provide such help. The aim of this study was to culturally adapt these guidelines for Chile and Argentina.

**Methods:**

A Delphi expert consensus study was conducted with two expert panels, one of people with lived experience of depression (either their own or as a carer; *n* = 26) and one of health professionals (*n* = 29). Overall, 172 statements from the English-language guidelines were translated and compiled into a questionnaire. Participants were asked to rate statements based on how essential or important those statements were for Chile and Argentina and to suggest new statements if necessary.

**Results:**

Data were obtained over two survey rounds. Consensus was achieved on 172 statements. A total of 137 statements were adopted from the English-language guidelines, whereas 35 new endorsed statements were generated from panel suggestions. There were similarities between the English-language guidelines and those for Chile and Argentina. The adapted guidelines did not include some of the items from the English-language guidelines related to commenting on a person’s strengths or making judgements about their character, and also incorporated new items related to the incorporation of sociocultural considerations as causes of depression and attention to inequities in mental health.

**Conclusions:**

The significant number of new items underscores the importance of undertaking a careful process of cultural adaptation. Further research on dissemination and incorporation of the guidelines into the Mental Health First Aid training course for Chile and Argentina is still required.

**Supplementary Information:**

The online version contains supplementary material available at 10.1186/s12888-023-04661-8.

## Background

Depression is one of the most common mental health problems in the general population. According to the latest Global Burden Disease study, depressive disorders have shown an increase in prevalence of 16% between 2010 and 2019, affecting 3.8% of the world’s population [1]. Depression is also one of the main causes of illness and disability worldwide. It has been ranked by the Institute of Health Metrics and Evaluation (IHME) as the second single largest contributor to global disability, contributing as much as 5.5% of all years lived with disability (YLDs) in 2019 [1].

The consequences of depression are frequently amplified by its comorbidity with other mental health problems, including substance use disorders [2] and anxiety disorders [3]. Depression is also relatively common in people with physical health problems, particularly in those with severe conditions such as stroke, acute coronary syndrome and cancer, contributing to poor quality of life, greater functional impairment and higher mortality rate of people with these conditions [4, 5]. Depressive disorders also have a considerable economic impact [6], with women, older people, and people of lower socioeconomic status among the most affected groups [7]. In turn, having depression may also contribute to and exacerbate inequalities, [8], particularly in low-and middle-income countries [9].

In Latin America, prevalence rates of depressive disorders are relatively high. According to the latest Global Burden of Disease report, depressive disorders in this region contribute 7.8% of YLDs, with disability burden in Chile and Argentina at 8.1% and 7.8% of YLDs, respectively [10]. In terms of prevalence, according to the latest National Health Survey 2016–17, Chile reported one of the highest 12-month prevalence rates worldwide: 6.2% for major depressive disorders and dysthymia, with a large difference between men (2.1%) and women (10.1%) [11]. In Argentina in 2018, the 12-month prevalence rate of major depression was reported to be 4.2% [12].

### Mental health services in Chile and Argentina

In Chile, the *Programa Nacional de Detección, Diagnóstico y Tratamiento Integral de la Depresión,* an evidence-based public policy focused on the primary care system, guarantees universal access to mental health care. This program has been proven effective with positive clinical and psychosocial results [13, 14, 15]. Available data in Chile [16] show that, by 2022, 84% of the total population undergoing treatment for an affective disorder were being treated at the Primary Health Care level, showing the relevance of this public policy.

In Argentina, the federal government structure, with very limited central coordination across the 24 jurisdictions, offers limited access to countrywide evidence-based programs while the availability of healthcare and programs targeting individuals with depression varies widely [17]. Moreover, psychoanalysis is overwhelmingly more accepted by clinicians than cognitive-behavioral therapy [18], the non-pharmacological approach for the treatment of depression most accepted worldwide [19].

### The mental health treatment gap

Despite the availability of effective treatments and public programs, on average, only 13.7% of people in low-and middle-income countries receive treatment for depression [20]. In Latin America in 2016, the number of people with common mental disorders who required treatment but did not receive it (treatment gap) was 74.7% [9]. In Chile, the latest available data showed a treatment gap for depressive disorder above 80% and only approximately 6.8% of people who met the criteria for a diagnosis of depression received services under the above-mentioned Depression program [11]. In Argentina, a 2008 study in the Metropolitan Area of Buenos Aires showed that only 23.1% of individuals with probable current clinically significant depression received specialty mental health care [21] while the latest epidemiological study in Argentina showed that 64.5% of individuals who met the criteria for a major depressive episode and 61% of those with dysthymia received no health care for their symptoms in the previous 12 months [12, 22].

The Covid-19 pandemic has widened the treatment gap. Available data in Chile show that the prioritization of Covid-related response reduced mental health care program delivery in the public system by about 58%, with a reduction of more than 38% for new confirmed cases of depressive disorders [16]. These data point to a potential exacerbation of the mental health treatment gap post-Covid due to the deferral of ongoing treatments and the incidence of new cases triggered by the pandemic.

Reducing the duration of untreated depression is crucial. In a meta-analysis, Ghio et al. [23] found that reducing the duration of untreated first episode of depression by eight weeks led to a 70% greater probability of responding to treatment and 65% greater probability of achieving remission. The authors argued that a longer duration of untreated depression was associated with a poorer response to antidepressant treatment and a low remission rate, increasing the risk for chronicity [23]. In addition, some evidence suggests that, unlike other health conditions, as depression symptoms increase, people are less likely to seek treatment [24, 25], contributing to the severity of the treatment gap.

There are several possible explanations for the treatment gap (both in terms of help-seeking and adherence to treatment). These include service issues like insufficient, inadequate, and inequitable coverage and access to mental health services [20], which interact with individual decisions related to avoiding seeking help or openly refusing it because of the fear of exclusion, public and internalized stigma, low perception of the treatment need or lack of mental literacy related to the effectiveness of the treatments [26, 27, 28]. In Latin America, stigma is likely to be a key barrier to help seeking [29, 30, 31], and this may interact with culturally specific factors such as religiosity, familism, the importance of social bonds, traditional male socialization, interdependent orientation and cultural obligations There is some evidence that southern Latin Americans (including people from Chile and Argentina) may be less open to mental health help-seeking than central and northern Latin Americans in the U.S migratory context [32].

### Community-based support strategies and Mental Health First Aid

Successfully addressing the treatment gap requires a balanced care model that includes both hospital and community-based care, incorporating services that support people to self-manage their mental health [33]. There is evidence that community engagement improves health outcomes [34] and may help to reduce the mental health treatment gap by boosting the health workforce, adjusting health programs to particular local needs and assets, reducing stigma, improving mental health literacy and promoting professional help-seeking [26, 27, 35]. The Lancet Commission on Global Mental Health and Sustainable Development has proposed that the involvement of non-specialists in delivering mental health interventions offers an opportunity to address global mental health challenges in a context of remaining treatment gaps, not least because it also helps to highlight the voices of people with lived experience of mental health problems [36].

In Chile in the 1970s, the Intracommunity Psychiatry Program was an example of an effort to employ lay former community workers within health services [37], and funding has been renewed in recent years, both for the lay members of the community [38], and for non-specialist health workers through strategies such as the Mental Health Global Action Programme (mhGAP) [39]. In Argentina, during the 1960s and 1970s, psychoanalysis and community psychiatry dominated mental health care, with the latter involving community members collaborating with mental health workers on a range of projects, including those not directly related to mental health [40]. In the 1990s, the Province of Neuquén promoted training primary-care doctors to collaborate in the treatment of people with mental disorders, including depression [41]. More recently, additional interest in training lay workers [42] and non-specialist mental health care workers [43] has emerged in response to fulfilling the requirements outlined in the 2010 National Mental Health Law.

Internationally, Mental Health First Aid (MHFA) training is an example of a strategy to improve the general population’s mental health literacy and to provide early intervention in a community environment where health services may not be available [44]. As mental health problems such as depression are prevalent in the community, there is a high probability of coming into contact with someone who is experiencing symptoms, which in turn provides an opportunity for non-health professionals to provide help. On this basis, Kitchener and Jorm [45, 46] developed the MHFA training courses to instruct people how to properly support someone who is developing a mental health problem or is in a mental health crisis. The training incorporates an action plan involving the following steps: 1) Assess risk; 2) Listen non-judgmentally; 3) Give reassurance and information; 4) Encourage appropriate professional help; and 5) Encourage self-help and other support strategies. This support is provided until the crisis is resolved, professional help is available or the situation resolves. MHFA has been widely disseminated, having spread to over 25 (mostly) high-income countries. A 2018 systematic review and meta-analysis of randomized controlled trials of MHFA training showed reductions in stigma and improvements in mental health literacy, and helping behavior up to six months after the training [47].

The content of the MHFA training course has been informed by guidelines developed using the Delphi expert consensus method with expert panels of health professionals and also with people with first-hand lived experience, either their own or as a caregiver. This gives greater external validity to the guidelines and offers the opportunity to incorporate practice-based evidence. It is an appropriate approach when experimental studies are not feasible [48]. Moreover, as the responses of all panel members have equal weight, this method does not prioritize the views of any one person and it also allows for the estimation of the degree of agreement between groups whose opinions might differ on some issues, without prioritizing the opinions of one group over another. This is especially important when analyzing the opinions of people with lived experience of mental health problems [49]. However, the first Delphi expert consensus studies were conducted in high-income English-speaking countries and the suitability of the guidelines for Chile and Argentina is unknown. Studies to culturally adapt English-language guidelines to other countries, such as Sri Lanka, Brazil and China [50, 51, 52, 53, 54], have highlighted a number of differences to the English-language guidelines, pointing to the need to undertake a similar process for Chile and Argentina.

Therefore, this study aimed to use the Delphi expert consensus methodology to culturally adapt guidelines for lay members of the community interested in providing mental health first aid to someone with depression in Chile and Argentina.

## Methods

As with the series of Delphi studies for culturally adapting the MHFA Guidelines that have been conducted in other countries [50, 51, 55, 56], this study comprised the following four stages: (1) Round 1 survey development; (2) Expert panel member recruitment; (3) Data collection and analyses for the round 1 and 2 surveys; and (4) Guidelines development.

### Round 1 survey development

The first round questionnaire was developed by translating the statements that were approved for inclusion in the mental health first aid guidelines used in English-speaking countries to support a person who may be experiencing depression [57]. All 172 items of the original guidelines were translated into Spanish and reviewed by bilingual mental health professionals from Australia, Argentina and Chile to ensure a culturally pertinent translation.

The Round 1 survey was composed of nine sections: (1) Recognition of signs of depression (9 items), which included items related to the core symptoms of depression, risk factors and its observation; (2) Ways to approach people with depression (26 items), which included items on giving practical advice on how to approach to the person (3) Ways to deliver support (46 items), which included items about first time help intervention; (4) Effective communication (32 items), which included items about practicing verbal and non-verbal communication skills; (5) Possible difficulties while providing support (22 items), which included items about identifying issues in the mental first aider-supporter relationship and the ways to address it; (6) Seeking help (24 items), which included items on providing relevant and pertinent information; (7) Actions when help is refused (7 items), which included items about acting in these situations; and (8) Safety concerns (6 items), which included items about dealing with situations where possible harm is involved.

### Expert panel member recruitment

Health professionals with expertise in working with individuals with depressive disorders and people with lived experience (in themselves or as caregiver) were recruited by six members of the research team (MA, EL and SAG, Argentina; EE, IZ and TT, Chile). Panel one comprised lived experience experts. Panel two comprised health professional experts. Lived experience experts could include participants from peer support groups, people who received treatment in health services and/or their caregivers. Health professionals included members of different health disciplines who work in health provision (e.g., clinical psychologists, psychiatrists, and primary care workers) as well as researchers and decision-makers. A Spanish translation of the original invitation from the English-language guidelines was used to recruit participants. They were asked for their views on actions related to how to help someone who is experiencing a mental health problem or is in a mental health crisis ("brindar sus opiniones sobre las acciones relacionadas con la forma de ayudar a alguien que está desarrollando un problema de salud mental o que se encuentra en una crisis de salud mental "). A broad definition of "person who may be experiencing depression" (“persona que puede estar experimentando depresión”) was adopted, without further specification. Recruitment was done through digital posts on the participating universities' social networks and by snowball sampling. In addition, members of the research team distributed information about the study to their personal contacts, asking them to communicate it to others with the required expertise. As with our previous study designs [55], the following criteria had to be met for a person to be eligible for the study.

- Health professional expert panel- with more than four years of experience working as a healthcare professional with expertise in depression. Eligible types of professions included, but were not limited to: general practitioners, nurses, occupational therapists, psychiatrists, psychologists, or social psychologists.
- Lived experience expert panel- self-identified as having experience with depression or caring for a person with depression.
- Aged 18 years old and above.

This study was developed during the Covid-19 pandemic, so participants provided informed consent by email, WhatsApp (a free US platform widely used for instant messaging between cell phones) and/or Google Forms. They signed the informed consent form with an image of their signature along with that of a witness.

### Data collection and analysis

Data for the first round was collected between March 09, 2020 and October 29, 2020. Data for the second round was collected between February 10, 2021 and May 05, 2021. The first-round surveys were conducted both on paper and online through Qualtrics software. The second-round surveys were conducted online only due to mobility restrictions due to the COVID-19 pandemic.

Using the same methodology as our previous studies [55], the surveys collected participants' ratings of a set of statements on a 5-point Likert scale (1 = essential, 2 = important, 3 = unsure, 4 = not important, 5 = should not be included), choosing how important they considered the inclusion of each statement in the final mental health first aid guideline for depression in Argentina and Chile. Items were immediately selected for the final guideline if at least 80% of the participants in both panels rated it as "essential" or "important". Meanwhile, statements rated as "essential" or "important" by 70.0—79.9% of the participants of at least one panel in the Round 1 survey were resubmitted in Round 2 for re-rating. Statements rated as "essential" or "important" by less than 70% of participants from at least one panel were immediately excluded from the final guideline. In Round 2, recommendations with an acceptance rate of at least 80% or more by one panel and at least 75% or more by the other panel were selected for the final guideline.

In the first-round survey, at the end of each subsection or after each 10 items (whichever came first), open-text response boxes were presented to allow participants to comment or suggest new recommendations that they felt were important to incorporate into the final guidelines. MA and TT elaborated new items based on the suggestions from the first round. These new items were reviewed again with NR before being included in the second round to ensure that they were actionable and clear and different from those already presented. Additionally, items that did not receive at least 80% support but were the subject of suggestions related to language or need for clarification were reformulated and included in the second round for re-rating.

Spearman's correlation coefficient was estimated for the association analysis between the approval ratings of the professional and consumer panels. SPSS version 25 software was used.

### Guidelines development for Chile and Argentina

EE and MA consolidated the recommendations from the two rounds of surveys into a preliminary guideline document. The rest of the team reviewed this draft version and made some comments. In parallel, this manuscript was sent to a small number of participants who explicitly expressed special interest in reviewing the draft guidelines. No criteria for selection were used and every expert who volunteered to review the draft received a copy; only minor changes that would not contradict the results of the Delphi process were included at this point.

### Ethical approval

The study received ethical approval from the University of Melbourne (in Australia), the University of Palermo (Argentina) and the University of Chile (Chile) (see below).

## Results

**Fig. 1:**
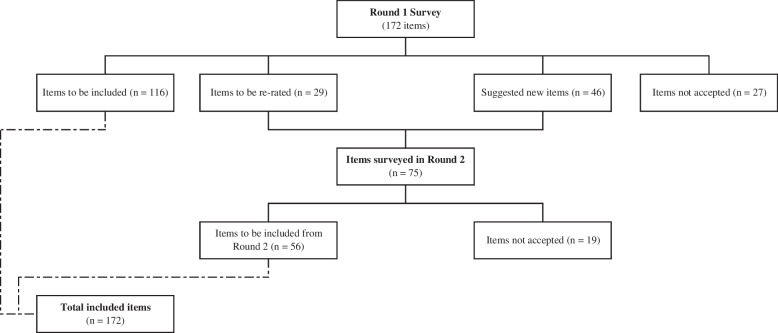
Number of statements included, re-rated and excluded in the two survey rounds

Figure 1 shows the overall process of including the statements through the two rounds.

### Round 1

A total of 55 participants completed the Round 1 questionnaire. The professional panel distribution (*n* = 29) had a minor difference between Chile (*n* = 12) and Argentina (*n* = 17) and included 14 psychologists, 11 psychiatrists, two general practitioners, one medical technologist, one social worker and one unspecified health professional. The average years of experience as a health professional was 28.0 years, with 58.6% females (*n* = 17) and 41.3% males (*n* = 12).

The lived experience panel (*n* = 26) had more Argentinian participants (*n* = 16) than Chilean (*n* = 10). Fourteen were consumers and ten were caregivers and/or relatives. Of those who identified themselves as consumers in their primary role, two were also carers; and of those who identified themselves as carers in the primary role, two were also consumers. A total of 61.5% were females (*n* = 16) and 38.5% were males (*n* = 10). Two experts, one from Argentina and one from Chile, who had been invited as part of the health professional panel also self-identified as consumers. Conversely, two experts, one for each country, who had been invited as part of the lived experience group, also identified themselves as health professionals. See Table 1 for a summary of the sociodemographic characteristics of participants.

**Table 1:** Sociodemographic characteristics of participants

Participant characteristics				
	First round *n* = 55		Second round *n* = 47	
	n	%	n	%
**Age**				
18–24	5	9.1	4	8.5
25–34	6	10.9	4	8.5
35–44	11	20.0	8	17.0
45–54	11	20.0	10	21.3
55–64	20	36.4	20	42.6
65 +	2	3.6	1	2.1
**Educational level**				
Primary	1	1.8	0	0.0
Secondary / high school	6	10.9	2	4.3
Technical training	7	12.7	6	12.8
Graduate (licenciate/bachelor)	18	32.7	15	31.9
Postgraduate (master/doctorate)	23	41.8	24	51.1
**Sex**				
Female	33	60.0	28	59.6
Male	22	40.0	19	40.4
**Professionals**	29	52.7	24	51.1
Psychologists	14	25.5	15	31.9
Psychiatrists	11	20.0	8	17.0
General practitioners	1	1.8	0	0.0
Medical technologists	1	1.8	0	0.0
Social workers	1	1.8	1	2.1
Unspecified health practitioner	1	1.8	0	0.0
**Source of experience (lay panel)**	26	47.3	23	48.9
Family experience or peer support experience	10	18.2	7	14.9
Own experience	16	29.1	16	34.0

Out of the 172 items included in the Round 1 survey, 116 items (67%) were endorsed as *essential* or *important* by 80% or more of the experts in both panels. Another 29 items (17%) required re-rating in Round 2, and 27 (16%) items were rejected (Fig. 1). Overall endorsement rates were 77.9% for the lived experience panel and 79.7% for the health professional panel, showing some preliminary consistency. Fifteen items (8.7%) were endorsed by one panel and rejected by the other panel, implying a good level of agreement between panels.

### Round 2

The Round 2 questionnaire included 29 items to be re-rated and 46 new items suggested in Round 1 (Fig. 1). A total of 47 participants completed Round 2, with 24 participants from the health professional panel (response rate of 82.7%) and 23 participants from the lived experience panel (response rate of and 82.1%). No new participants were added in Round 2. Out of the 75 items rated in Round 2, 75% (*n* = 56) were endorsed by both panels and 25% (*n* = 19) were rejected.

### Differences between the Spanish-language guidelines for Chile and Argentina and the English-language guidelines

In total, across the two rounds, 172 items were endorsed, and 46 items were rejected. When comparing the English and Spanish guidelines, it was noted that 35 statements (20.3%) included in the English guidelines were not accepted by the Argentinian and Chilean experts. Among the 46 new items, the proportion of new suggested items rejected (23.9%) was slightly higher than the original items rejected (20.3%). Overall, 35 new items were added. See Supplementary file 3 for key differences between the English-language guidelines and those for Argentina and Chile.

The group of rejected statement included (a) many items recommending self-help (i.e., encouraging to use them or ask for past useful self-help strategies), (b) some attitudes for helping people who are at risk of self-harm or harm to others (i.e., to respect the person’s right to not seek help or ask the person to take steps to get help), (c) non-verbal skills in help-offering (i.e., sitting alongside the persons rather than directly opposite them or using minimal prompts to keep the conversation going), (d) encouragement for people to talk about their problems or seek help (i.e., recommending other available service like telephone counseling, encouraging the person to talk about feelings and thoughts or reinforcing an optimistic view if the person judge themselves too harshly), and (e) other items from different sections of the guidelines (i.e., do not adopt an over-protective attitude or discussing what is culturally appropriate for them).

Overall, the item with the lowest endorsement was “The first aider should encourage the person to do a list of questions to discuss with the health professional at their first appointment”, which had an average rating of 41.0% (30.8% lived experience – 51.7% professional panel). With 51% average endorsement (46.2% lived experience panel – 55.2% professional panel), the statement “The first aider should use the following nonverbal skills to reinforce their nonjudgmental communication:—Sit next to the person and at an angle to them, rather than directly in front of them” was the second-lowest rated. Other rejected items included the following: The first aider should: “ask the person if what they are doing is helpful, and what else they could do to help”, “should know that recovery, for the most part, should be person-led” or “must respect the person’s interpretation of the signs and symptoms”.

On the other hand, the new statements added comprised considerations on the social determinants of mental health; considerations for minority or disadvantaged groups; the limitations of help mediated by virtual platforms; clues about non-verbal aspects of help and considerations about the first aider's expectations of help, especially in the face of circumstances such as the person's anger or embarrassment.

In the first section, experts added statements about social stressors (such as economic stress) which could lead to chronic depressive symptoms, identifying vulnerable groups (including members of the LGBTIQ + community) which may require particular consideration. In terms of first aid provision, experts included the recognition of the limits of self-help and the importance of personal meeting, the respect for the autonomy of the person in the decision to seek and receive help and considering that there are people who need to be accompanied in silence. Suggestions also comprised a range of actions including asking the person why they were upset, trying to politely redirect the conversation and reiterating their willingness to help, taking a break in the conversation and asking the person how they want to continue. Finally, the experts included some new suggestions, such as the necessity of the first aider considering in each case if they are suitable to provide first aid, learning to know their limits, asking for additional help, and being clear with the person if and when it is time to stop assisting them. They also included items about the need to be part of a support network for a more comprehensive response.

See Supplementary file 3 for a list of the statements excluded from the original guidelines and new items added in the final guidelines.

### Differences between the lived experience and health professional panels

Over both rounds, the level of agreement between panels was high (with Spearman *r* = 0.63 in Round 1 and *r* = 0.59 for total items rated *p* < 0.01). Overall, for 75.5% (*n* = 130) of the statements there was less than a 10% difference in the percentage of panel members endorsing those items, including 8.7% (*n* = 15) of the items with complete agreement in both panels (i.e., 100% of the members of both panels endorsing the item). On the other hand, there were 6.9% of items (*n* = 12) where disagreement between panels was 20% or greater and only 1.7% of items with disagreement greater than 30%. The mean difference in inter-panel approval for the 172 items was 7.4%.

In round 2 the correlation between panels was lower (Spearman *r* = 0.47 *P* < 0.01). Two thirds of items had less than 10% difference in the percentage of members of panels endorsing those items, including 13.3% of items with an absolute agreement. Disagreements of 20% were found in 9.3% of items, including 4% of items with differences above 30%. The average difference between percentage approval for all 75 items was 9% between panels. The correlation between two panels, considering all statements rated (in case of re-rated statement, taking the last score), was *r* = 0.59 (*p* < 0.01).

The greatest differences in Round 1 included items related to the sources of help, the self-help strategy. For example the statement “If the person finds it difficult to discuss their thoughts and feelings openly, the first aider should let the person know about available services where they can talk to someone else, e.g. a telephone counselling service”, had a 34.6% difference (65.4% lived experience panel – 100% professional panel) and the statement “If the person is interested in self-help strategies, the first aider should: encourage them to consult reputable sources about what is most likely to be helpful, e.g. a health department-sponsored website” had a 29% difference (53.8% lived experience panel – 82.8% professional panel),

There were also differences in recommendations about reminding the person about their strengths. For example, the statement “If the person says that they feel they are a weak person or a failure, the first aider should let the person know that they do not believe that the person is weak or is at fault” had 41.6 percentage points difference in ratings (95.8% lived experience panel – 54.2% professional panel), the greatest difference in all rated statements. In addition, the item “If the person says that they feel they are a weak person or a failure, the first aider should let the person know that they don’t think less of them as a person” had 37.5 percentage points of endorsement difference (100% lived experience panel – 62.5% professional panel), the second greatest difference. Similarly, the statement “If the person judges themselves too harshly, remind them of their strengths and virtues” had 36.8 percentage points of endorsement difference (88.5% lived experience panel vs 51.7% professional panel).

There were also considerable differences in the endorsement of the item “The first aider should learn more about the depression by: seeking advice from people who have experienced and recovered from depression” had a 33.3% difference (88.5% lived experience panel vs 55.2% professional panel). Related to that, the statement “The first aider should consider inviting the person to jointly search for information and appropriate ways to deal with what they are experiencing” had 37.5% percentage difference (58.3% live experience panel vs 95.8% professional panel),

See supplementary file 1 for details of the ratings of statements by round and panel, and supplementary file 2 for the final guidelines text in Spanish.

## Discussion

The present study aimed to use the Delphi expert consensus method to culturally adapt guidelines for community members wishing to provide mental health first aid to someone with depression symptoms in Chile and Argentina. This was achieved by a two-round Delphi survey, involving mental health professionals, people with their own lived experience and carers. The final guidelines comprised 172 statements endorsed by 55 professionals and lived experience experts in the first round and 47 in the second round.

### Differences between health professional and lived experience panels

Notable differences between panels were largely seen in items relating to judgements about a person’s character (e.g., being a weak person or a failure or the necessity of the first aider reminding them about their strengths). The lived experience panel strongly endorsed these items as being important to provision of first aid for depression, but due to lower levels of endorsement by professionals, they were not included in the final guidelines. Among these, items related to sharing an opinion that reinforces a positive view of the person when they were judging themselves too harshly, were explicitly rejected by health professionals. These differences may reflect the influence of health professional training on the conception of mental health first aid. Health professionals may be more likely to focus on pathology and may also have viewed such statements as being inappropriate in clinical practice due to the risk of making overly personal comments or trivializing the person’s problems [58, 59], while for people with lived experience aspects of strength and resilience were more likely to be valued and they may have viewed such comments as helpful in the first aid process. This perspective contrasts with some components of the *Recovery* framework, like micro-affirmations, “small things” and other practices where professionals can share their own perceptions and feelings about a person’s recovery process with the person [60, 61].

Another group of statements for which ratings diverged, were those related to the role of people with lived experience, both as a source of knowledge and as a part of intervention itself, possibly reflecting views about the legitimacy of non-specialists knowledge and skills, where, even though there is increasing research in this field in LMICs [62], significant barriers to its development remain [63, 64].

Finally, a third group of statements with large differences between panels, pointed to low levels of recommendation by the lived experience panel of some sources of help, such as telephone counselling service or health websites. This may point to lower confidence in these sources of help, showing the importance of including health communication strategies in the promotion of help seeking, especially considering the well-established effectiveness of technology-mediated interventions in improving mental health literacy [65].

As a whole, these differences can provide some keys to understanding the major contrasts in perspectives between professionals and people with lived experience. Further research should investigate population mental health literacy in Chile and Argentina, including exploration of beliefs about help seeking, self-help actions and key population groups to target [66]. This would further assist in development of interventions to improve knowledge, attitudes and behaviours such as MHFA training.

### Comparison with the guidelines for English-speaking: More substantial changes than other countries

Out of 172 English-speaking original statements, 35 were rejected and 35 new suggested items were included. Even though most of the original statements were endorsed (79.6%), this proportion represents a lower percentage than those recently endorsed in China (92%) [50] and Sri Lanka (92%) [51], countries where the same guidelines have been recently adapted. In the Sri Lankan adaptation, 9 new items were included and 19 were rejected. In the Chinese adaptation, 12 new statements were included and 14 were rejected. These differences show a potentially greater need for cultural adjustment in Chile and Argentina than in these countries.

Some of the rejected items, including those relating to the involvement of people with lived experience, may imply that there is still limited support for non-professionals to be involved in any therapeutic process[64]. In addition, rejections of statements about non-verbal communication pointed to the importance of considering cultural appropriateness when delivering mental health first aid, and are likely to be important in development of MHFA training for Chile and Argentina [67]. This is supported by the findings of similar studies in China and Sri Lanka [50, 51], where some items rejected in Chile and Argentina’s guidelines were endorsed. An example is the consistent Chinese approval of statements related to always respecting what the person allows, while Chilean, Argentinian and Sri Lankan experts tended to agree with a more directive role by the first aider.

On the other hand, new statements included some specific considerations on social determinants of mental health, such as economic stress and being part of a marginalized group, pointing to a view in which risk exposure and vulnerability partly explain health outcomes [68]. On the other hand, the attention to the limitations of help mediated by virtual platforms could be given extra emphasis by Covid-19 pandemic (reflecting the period in which the present study was conducted), which has raised awareness of health inequity and has prompted people to recognize the limits and contributions of virtual assistance [69]. Finally, special statements were added in the face of circumstances such as the person's anger or embarrassment. It is possible that this relatively high level of attention to the potential of the first aider to cause distress is related to an environment of increased tension and violence in both countries [70], which may lead people to resist offers of help [71]. While these suggestions were not entirely new or contradictory to statements in the English-language guidelines, they did outline more specific guidance, pointing to the relatively greater emphasis on these actions for Chilean and Argentinian culture.

### Strengths and limitations

A key strength of this study is a research design that gives equal weight to the views of health professionals and people with lived experience. This is relevant considering that the objective was to culturally adapt the recommendations, an issue that is unlikely to be achieved only with input from professionals.

In terms of limitations, participants were mainly from metropolitan areas, which limits the generalisability to rural areas. However, inclusion of participants from both Chile and Argentina, supports the case for generalisability to other Spanish-speaking countries, particularly metropolitan areas. Another limitation relates to the relatively high literacy and levels of education of the participants, as the survey required access to a computer or phone, which may only be available to more highly educated participants. A final limitation is that full back-translation was not performed once the original statements were translated into Spanish, which could have ensured greater reliability and fidelity to the original items.

## Conclusion

A Delphi expert consensus study involving health professionals and people with lived experience was used to adapt the mental health first aid guidelines for depression for Chile and Argentina. The adapted guidelines did not include some of the items from the English-language guidelines related to commenting on a person’s strengths or making judgements about their character and also included new items related to the incorporation of sociocultural considerations as causes of depression, and attention to inequities in mental health. Further research on dissemination and uptake of the guidelines in Chile and Argentina is necessary as well as research into incorporation of the guidelines into MHFA training for these countries.

## Supplementary Information

**Additional file 1: Supplementary file 1, table 1.** Items rated in Round 1.

**Additional file 2. **Depression MHFA Guidelines for Chile and Argentina.

**Additional file 3: Supplementary file 3.** Differences between the English-language mental health first aid guidelines for depression and those for Argentina and Chile.

## Data Availability

The data supporting our findings is attached as Additional file 1, which contains all the statements that were presented to the panels and their endorsement rates.

## Abbreviations

MHFA: Mental Health First Aid
LMICs: Low- and middle-income countries
