# Supplementary material for: Development of mental health first-aid guidelines for depression: a Delphi expert consensus study in Argentina and Chile
Source: BMC Psychiatry. 2023 Mar 14;23:161. doi: 10.1186/s12888-023-04661-8 (PMC10013290; doi:10.1186/s12888-023-04661-8)
Supplement: Supplementary file 2 — Additional file 2. Depression MHFA Guidelines for Chile and Argentina. [file 12888_2023_4661_MOESM2_ESM.pdf]

MENTAL HEALTH FIRST AID
Chile - Argentina

CÓMO BRINDAR AYUDA A UNA PERSONA CON DEPRESIÓN:

RECOMENDACIONES DE PRIMERA AYUDA EN SALUD MENTAL

1. ¿Cómo sé si alguien está experimentando una depresión?

Para poder ayudar a alguien que está experimentando depresión, debes saber cuáles son sus signos y síntomas. La experiencia de la depresión puede variar en gravedad, por ejemplo, desde la sensación de irritabilidad a sentirse inclinado a quitarse la vida. Cada individuo es diferente y no todos quienes experimentan depresión mostrarán sus signos y síntomas típicos. Algunas personas que se han recuperado de la depresión pueden tener una recaída de sus síntomas. Incluso si la persona ha tenido un episodio previo, no deberías asumir que sabrá cómo manejar el episodio actual.

Según el Manual Diagnóstico y Estadístico de los Trastornos Mentales Quinta Edición (APA, 2013)[1] , los siguientes son algunos de sus signos más comunes. Debe ser preocupante si estos persisten en el tiempo y afectan el funcionamiento de la persona:

• Un estado de ánimo deprimido.
• Pérdida de placer e interés en actividades que solían ser agradables.
• Falta de energía y cansancio.
• Sentirse inútil o culpable cuando no es realmente culpable.
• Pensar mucho en la muerte o en el suicidio.
• Dificultad para concentrarse o tomar decisiones.
• Moverse con más lentitud o, a veces, agitarse y ser incapaz de calmarse.
• Tener dificultades para dormir o a veces dormir demasiado.
• Pérdida de interés por la comida o, a veces, comer demasiado.

Según la Guía Clínica la Depresión en mayores de 15 años del Ministerio de Salud de Chile (2013)[2], entre los principales factores de riesgo para la depresión podemos encontrar :

• Presencia de enfermedades crónicas, como el cáncer, diabetes, problemas cardíacos o el VIH han demostrado incrementar el riesgo de desarrollar un trastorno depresivo.
• La fatiga crónica ha mostrado asociación con la presencia de trastornos depresivos, distimia y trastorno bipolar.
• Situaciones de violencia intrafamiliar.
• Historia familiar o antecedentes personales de depresión.
• Eventos traumáticos o eventos vitales estresantes.

Referencias:

[1] Asociación Americana de Psiquiatría (2016). Manual diagnóstico y estadístico de los trastornos mentales. Quinta Edición. Ed. Panamericana.

[2] Ministerio de Salud de Chile (2013). Guía clínica AUGE Depresión en personas de 15 años y más. Disponible en www.minsal.cl

MENTAL HEALTH FIRST AID
Chile - Argentina

CÓMO BRINDAR AYUDA A UNA PERSONA CON DEPRESIÓN:

RECOMENDACIONES DE PRIMERA AYUDA EN SALUD MENTAL

Debes saber que hay factores estresantes que pueden hacer crónicos los síntomas, tales como problemas médicos de salud, problemas económicos o problemas familiares. Es importante que conozcas las características de los grupos minoritarios, excluidos o vulnerables, mujeres, niños y personas mayores, que pueden presentar características o síntomas particulares de los trastornos depresivos.

Es importante que aprendas más sobre la depresión, leyendo o escuchando las experiencias de depresión de otras personas y también leyendo sitios web o libros de información de buena reputación.

¿Cómo saber si la información es fiable y precisa?

Hazte las siguientes preguntas para determinar si una fuente de información es fiable:

• ¿Es el autor conocido y respetado en el campo de la depresión o la salud mental?

• ¿Procede la información de una institución creíble, por ejemplo, una clínica u organización sanitaria conocida y respetada, un departamento gubernamental o una universidad?

• ¿Qué antigüedad tiene la información? (Una buena regla general es buscar materiales publicados en los últimos diez años).

• ¿Han incluido los autores una fecha de la información publicada? Si no es así, no podrás saber cuál es la antigüedad de la información.

• ¿Cuál es el dominio del sitio web? Busca sitios con dominios .edu, .int y .gov.

• ¿Contiene errores ortográficos o una gramática deficiente? Esto indica que el sitio puede no ser creíble.

• ¿Es un blog, una publicación de Facebook o un sitio de autoría propia?

Puede que no sean fiables. Siempre trata de verificar la información obtenida de estas fuentes.

CÓMO BRINDAR AYUDA A UNA PERSONA CON DEPRESIÓN:

RECOMENDACIONES DE PRIMERA AYUDA EN SALUD MENTAL

2. ¿Cómo debo acercarme a alguien que está experimentando depresión?

Si sospechas que una persona está experimentando depresión, no debes ignorar ningún signo o síntoma que hayas notado, ni asumir que la depresión simplemente desaparecerá. Tampoco debes asumir que necesariamente los síntomas (por ej., quejas respecto de su salud física) se deben a la depresión, ni que cualquier signo o síntoma que hayas notado (por ej., irritabilidad o tristeza) significa que la persona está experimentando depresión.

Si estás preocupado/a por alguien que pueda estar deprimida/o, debes decirle que te importa y que quieres ayudarle. Si la persona dice que se siente triste o deprimida, debes preguntarle cuánto tiempo hace que se siente así. Demorar el acercamiento, o no aprovechar una oportunidad para identificar tempranamente un problema de depresión, puede llevar a la persona a tener mayores problemas en el futuro.

Muchas personas que sufren depresión también pueden verse afectadas por otros problemas de salud mental, como ansiedad o problemas de uso de sustancias. Si notas cambios en el estado de ánimo, el comportamiento o el funcionamiento diario de alguien que conoces, debes acercarte y conversar acerca de sus preocupaciones. Permitir que la persona hable sobre cómo se siente, puede ayudarla a sentirse mejor, no peor.

Debes prepararte para una gama completa de reacciones (por ejemplo, alivio, indiferencia, ira) cuando te acerques a la persona para conversar con ella acerca de su depresión. Es importante elegir el momento adecuado, cuando tanto tú como ella tengan tiempo para hablar y se sientan cómodos, en un lugar que sea privado, y donde se pueda conservar la confidencialidad de lo que se hable.

Algunas personas que se han recuperado de la depresión pueden tener una recaída de sus síntomas. Incluso si la persona ha tenido un episodio previo, no deberías asumir que sabrá cómo manejar el episodio actual.

Es bueno que consideres si eres la mejor persona para acercarte a hablar, o si alguien más podría ser más apropiada/o. Debes preguntarle a la persona si está dispuesta a hablar contigo, o si prefiere hablar con alguien más y, si no se siente cómoda hablando contigo, debes alentarla a que hable con alguien más acerca de cómo se siente. Una persona puede sentirse agotada o invadida si le preguntas demasiado insistentemente acerca de cómo se siente.

Antes de ofrecer información a la persona, debes considerar su nivel educativo y su capacidad para comprender la información, así como que los recursos sean precisos y apropiados para su situación. Tampoco debes abrumar con demasiada información o demasiados recursos. Es importante explorar el contexto sociocultural de la persona antes de sugerir acciones que puedan no ser viables. No mientas ni des excusas por el comportamiento de la persona, ya que esto puede llevarla a demorar su búsqueda de ayuda.

Debes advertir la importancia de los encuentros personales para ayudar a la persona, y de las limitaciones de mensajes u otras formas de contacto diferido (por ej., los mensajes de whatsapp). De todas formas, evalúa la pertinencia de utilizar plataformas digitales para comunicarte con la persona cuando los encuentros no sean posibles.

3. ¿Cómo puedo dar apoyo?

Es importante tener en cuenta que la situación y las necesidades de cada persona son únicas. Debes respetar los sentimientos, valores personales y experiencias de la persona como válidos, incluso si son diferentes de los tuyos, o no estás de acuerdo con ellos.

Debes respetar la autonomía de la persona mientras consideras en qué medida puede tomar decisiones por sí misma, y si corre el riesgo de hacerse daño a sí misma o a los demás. Las conversaciones que tengas con la persona deben permanecer en privado, a menos que estés preocupado/a de que pueda hacerse daño a sí misma o a otros.

Recuerda que la depresión es una enfermedad y que no es culpa de la persona que esté experimentando depresión. Debes reconocer cualquier esfuerzo que esté haciendo para mejorar y aceptar a la persona tal como es, manteniendo expectativas realistas para ella.

MENTAL HEALTH FIRST AID Chile - Argentina

CÓMO BRINDAR AYUDA A UNA PERSONA CON DEPRESIÓN:

RECOMENDACIONES DE PRIMERA AYUDA EN SALUD MENTAL

Una persona deprimida puede verse abrumada por temores irracionales. No debes presionarla para que haga actividades que sienta que son demasiado para ella. Considera que algunas actividades cotidianas (como salir a la calle, interactuar con gente u ocuparse de la casa) pueden parecerle abrumadoras. Ten presente en todo momento que las personas no están "fingiendo", siendo "vagas", "débiles" o "egoístas" al experimentar una depresión. Hazle saber que no es débil ni es un fracaso tener una depresión y que eso no la vuelve menos valiosa como persona, reforzando que también las personas fuertes y capaces pueden deprimirse.

Se paciente y tolerante.

Es fundamental que seas paciente, persistente y alentador al apoyar a alguien con depresión, entendiendo que la persona necesita apoyo y comprensión adicionales. Debes ofrecer amabilidad y atención, incluso si no es recíproco. Es probable que tu apoyo tenga un impacto positivo, incluso si no es así en un comienzo. Debes ser consistente y predecible en tus interacciones. A menudo, simplemente tomarse el tiempo para hablar o estar, le hace saber a la persona que a alguien le importa.

Algunas cosas que podrías considerar decirle:

• Que no está sola/o en esto

• Que estás ahí para ayudar

• Que aunque no seas capaz de entender exactamente cómo se siente, te importa y quieres ayudarla

• Que puede que no lo crea ahora, pero la forma en que se siente, mejorará

• Que con tiempo y tratamiento se sentirá mejor

• Que aunque su experiencia es muy personal y dolorosa, no está sola.

• Que la depresión no es un tema moral ni de capacidad, sino que es un tema de salud

No tiene sentido simplemente decirle a la persona que se mejore, ni recomendarle que se mantenga más ocupada o salga más. No debes trivializar sus experiencias diciendo que "es importante sonreír", que "le ponga un poco de voluntad" o que "se tome las cosas con calma". Evita decir frases como "Todo está en tu cabeza" o "No puedo hacer nada para mejorar tu situación". No menosprecies ni descartes los sentimientos de la persona esforzándote por decir algo positivo como "No me parece tan malo".

Respeta la autonomía de la persona.

Debes respetar la autonomía de la persona salvo que corra riesgos de hacerse daño o hacerles daño a terceros, y no esté en condiciones de decidir por sí misma. Previa aceptación de la persona, debes tratar de involucrar a otros significativos (como familiares, allegados, o amigos), a menos que la situación sea de tanta gravedad que debas considerar involucrar a otros por más que no cuentes con la aceptación de la persona.

Deberías familiarizarte con aspectos cognitivos típicos de la depresión, por ejemplo pensamientos negativos, y saber cómo aproximarte cuando estos estén presentes. También ten en cuenta que si lo que dices no sintoniza con el estado de ánimo de la persona, puede ser percibido como una falta de empatía o no estar siendo comprendida.

Junto con acompañar a buscar ayuda profesional, puedes sugerir a la persona realizar actividades y evitar aislarse, aunque al principio no las disfrute. Especialmente cuando se está sintiendo mal, es positivo reforzar todas aquellas actitudes o acciones que muestren una mejoría o sean un logro. No deberías restringirte a hablar únicamente de aspectos relacionados con la depresión si es que la persona está dispuesta a hablar de otros temas.

Es importante formar parte de una red y contar con una red de apoyo personal para poder acompañar mejor a personas con depresión.

Es importante transmitir calidez, seguridad y tranquilidad. Debes transmitir la idea de que el proceso de recuperación es gradual, y que a veces hay retrocesos antes de seguir avanzando. Llegado el momento de dejar de acompañar a la persona, debes haberla preparado primero.

En el caso que la persona sea adolescente, es importante que estés entrenado en la problemática específica (así como en la interacción con madres, padres u otros/as cuidadores/as) y con conocimientos básicos de las normativas legales vigentes en tu zona.

CÓMO BRINDAR AYUDA A UNA PERSONA CON DEPRESIÓN:

RECOMENDACIONES DE PRIMERA AYUDA EN SALUD MENTAL

Se honesto y transparente.

Con todo, es importante que seas claro y coherente en cuanto al apoyo que puedes y que no puedes ofrecer. No debes hacer promesas que no puedes cumplir y debes ser honesto acerca de las limitaciones de tu función como asistente o agente de primeros auxilios (por ej., que no eres consejero/psicólogo), siendo honesto sobre qué ayuda puedes y estás dispuesto a ofrecer. Debes resistir el impulso de tratar de curar la depresión de la persona, evaluando, en cada caso, si eres idóneo para prestar primeros auxilios a esa persona. Es importante aprender a conocer tus límites y pedir ayuda cuando los has excedido.

4. Comunicación efectiva

Para asegurar una comunicación efectiva en primera instancia, deberías preguntarle a la persona si algo le ha sucedido recientemente que pueda estar contribuyendo a cómo se siente. Anímala a hablar sobre sus pensamientos, sentimientos, síntomas y cualquier otro problema que tenga, cuando esté lista. No debes presionarla para que hable de inmediato.

Si ocurre que a la persona le resulta difícil hablar de sus pensamientos y sentimientos abiertamente, debes sugerir una actividad que pueda facilitarles la conversación (p.ej. tomar una taza de té, tomar mate o salir a caminar). Si no tiene la energía o la inclinación para hablar acerca de cómo se siente, no la presiones para que lo haga.

Debes saber que las actitudes claves implicadas en la escucha sin prejuicios son la aceptación, la autenticidad y la empatía. Es más importante que realmente te preocupes por la persona, más que el hecho de decir todo lo que "se debe decir".

Debes transmitir autenticidad mediante el uso de un lenguaje corporal que coincida con tu comunicación verbal, p. ej. decirle que aceptas y respetas sus sentimientos, mientras mantienes una postura abierta y contacto visual apropiado. Demuestra empatía mostrándole que realmente es escuchada y entendida, p. ej. diciendo: "lo que estás pasando debe ser difícil" o "entiendo que no la estás pasando bien".

Es importante que adoptes una actitud de aceptación de la persona al:

• Reservarte cualquier juicio que hayas hecho sobre la persona o sus circunstancias, p. ej, si sientes que está siendo floja, no debería expresar esto.

• Elegir palabras cuidadosamente para evitar ofender, p. ej. no aplicar etiquetas que puedan encontrar estigmatizantes, como "enfermos mentales"

Es fundamental que dejes de lado tus creencias y controles tus reacciones negativas (si te surgieran). Céntrate en las necesidades de la persona a la que estás ayudando, haciendo preguntas abiertas para darle la oportunidad de decir lo que quiere, y escuchar atentamente, incluso si lo que está diciendo no es cierto o es inconducente. Debes escuchar sin juzgar.

Debes usar la misma terminología que usa la persona cuando habla de su experiencia, excepto si usa un lenguaje que no es de ayuda o que es estigmatizador, o que la terminología alternativa que uses pueda clarificar o facilitar la expresión de los sentimientos de la persona. No uses un lenguaje relacionado con un diagnóstico potencial cuando hables con la persona

Cómo ser un buen oyente

Ser un buen oyente implica mostrar que estás escuchando. Puedes usar las siguientes habilidades verbales para hacerlo:

• Hacer preguntas que demuestren que realmente te importa y quieres entender lo que dice.

• Verificar su comprensión al repetir lo que dijo y resumir hechos y sentimientos.

• Escuchar no sólo lo que dice, sino cómo lo dice (p. ej. su tono de voz).

• Ser paciente, incluso cuando la persona no se está comunicando bien, puede ser repetitiva o puede estar hablando más despacio y con menos claridad de lo normal.

• No ser crítico ni expresar tu frustración por no poder comunicarse bien.

• Evitar dar consejos inútiles como “Ponte / ponete bien” o “Anímate”.

• No interrumpir cuando está hablando, especialmente para compartir tus propias opiniones o experiencias.

• Evitar la confrontación, a menos que sea necesario para impedir que la persona cometa actos dañinos o peligrosos.

Además, puedes usar las siguientes habilidades no verbales para reforzar tu comunicación sin prejuicios:

• Presta mucha atención a lo que dice.

• Mantén un contacto visual con el que la persona parezca más cómoda.

• Ten en cuenta el lenguaje corporal de la persona, ya que esto puede proporcionar pistas sobre cómo se siente o qué tan cómoda se siente al hablar contigo.

• Observa con cuánto espacio personal se siente cómoda y respétalo.

• Mantén una posición receptiva o de cuerpo abierto (p.ej., no cruzar los brazos, ya que esto puede parecer defensivo).

• Evita los gestos que distraen (p.ej. jugar con un bolígrafo o mirar otras cosas), ya que podrían interpretarse como una falta de interés.

Ten presente que la persona misma puede tener actitudes estigmatizadoras hacia la enfermedad mental, y debes transmitir actitudes que sirvan de modelo para una mayor aceptación de los padecimientos mentales. Debes tratar de ver los comportamientos irritables o desagradables, si los hubiera, como parte de lo que está viviendo la persona y no tomarlos como algo personal. Si perteneces a su círculo, debes evitar decir frases como “no quisiste escuchar”, “te lo dije” o, “no me haces caso”. Si no perteneces a su círculo, intenta conocer y comprender a la persona para saber cómo relacionarte mejor con ella.

Considera que hay personas que necesitan que las acompañen en silencio y que a veces eso les permite expresarse espontáneamente. Debes estar preparado por si la persona reacciona con molestia, agresivamente, o no toma positivamente algo que le digas, o si siente vergüenza de hablar de sus problemas y adopta una actitud evitativa.

Estigma en salud mental

El estigma puede ser definido como una condición, atributo, rasgo o comportamiento que hace que quien lo porte, genere una respuesta negativa y sea visto como culturalmente inaceptable o inferior. El estigma en salud mental es la etiqueta negativa que se pone sobre las personas con problemas de salud mental, considerándoles peligrosos, débiles o inútiles, generando actitudes y actos discriminatorios de rechazo hacia ellos/as. Según Mascayano, Lips y Mena (2015)[3] el estigma hacia (o en) personas que presentan problemas de salud mental puede provenir de las instituciones, la familia, pero también de de las propias personas con problemas de salud mental, de manera internalizada o baja la forma de autoestigma.

Debido a los prejuicios y actos discriminatorios, es frecuente que las personas etiquetadas presenten bajo nivel de autoestima y menor calidad de vida, puedan evitar realizar consultas por salud mental en el sistema de salud, así como presentar baja adherencia a los tratamientos y una reducción significativa de sus redes sociales. Adicionalmente, presentan un acceso limitado a espacios laborales o educacionales normalizados, afectando el establecimiento de relaciones de amistad o de pareja. En suma, el estigma como interacción social es uno de los principales obstáculos para lograr la plena inclusión social de los individuos con algún padecimiento psíquico (Mascayano, Lips y Mena, 2015).

Referencias:

[3] Mascayano TF, Lips CW, Mena PC, et al. Estigma hacia los trastornos mentales: características e intervenciones. Salud Mental. 2015;38(1):53-58.

CÓMO BRINDAR AYUDA A UNA PERSONA CON DEPRESIÓN:

RECOMENDACIONES DE PRIMERA AYUDA EN SALUD MENTAL

5. Dificultades que puedes encontrar en la primera ayuda

Relacionadas con tu experiencia

Ayudar a alguien que está deprimido puede provocar en ti una respuesta emocional inesperada o imprevista. Si el comportamiento de la persona tiene un efecto negativo en ti, debes reconocer tus propios sentimientos y tratarlos por tu cuenta. No debes aceptar el abuso ni comprometer tu propia salud mental en el proceso de apoyo.

Si te sientes molesto o agotado después de ayudar a la persona, deberías:

• Encontrar a alguien con quien hablar sobre tus sentimientos sin compartir los detalles personales de la persona a la que estabas ayudando.

• Usar estrategias de autocuidado basadas en evidencia.

Algunas recomendaciones para cuidar tu propia salud mental

Puede ser bueno hacer cosas que mejoren tu propio estado de ánimo o tu salud mental después de ayudar a otras personas. Las actividades que se sabe que son útiles para mejorar el estado de ánimo y reducir la ansiedad incluyen comer bien, mantener unos hábitos de sueño regulares, practicar técnicas de relajación (por ejemplo, la relajación muscular progresiva), mantenerse físicamente activo, hablar con personas que te apoyen, hacer saber a otras personas cómo te sientes, programar actividades agradables (en particular, las que dan una sensación de logro) y hacer otras cosas que sabes que han sido útiles en el pasado[4].

Referencias:

[3] Morgan AJ, Jorm AF, Mackinnon AJ. Email-based promotion of self-help for subthreshold depression: Mood Memos randomized controlled trial. British Journal of Psychiatry 2012; 200:412-8.

[4] Morgan AJ, Jorm AF, Mackinnon AJ. Email-based promotion of self-help for subthreshold depression: Mood Memos randomized controlled trial. British Journal of Psychiatry 2012; 200:412-8.

Relacionadas con la persona que ayudas

Si la persona no quiere hablar, debes informarle que estás disponible para hablar cuando esté lista y, si no quiere hablar contigo, debes sugerir que hable con otra persona. Las pausas y los silencios están bien y, si bien puedes sentirte incómodo/a, la persona puede necesitar tiempo para pensar o encontrar las palabras adecuadas.

Si la persona se enoja durante la conversación, deberías:
• Mantener la calma.
• Reconocer la ira.
• No hacer suposiciones sobre la causa de su enojo.
• Considerar preguntar qué fue lo que le causó molestia.
• Intentar reencauzar el diálogo amablemente y reiterar tu disposición a ayudar.
• Considerar tomar un descanso en la conversación y preguntar a la persona cómo quiere seguir.
• En el caso de no poder controlar la situación, intenta tranquilizar a la persona y considera buscar apoyo.

Si ayudas a alguien de una formación cultural diferente a la tuya, deberías estar dispuesto a ajustar tus conductas verbales y no verbales. p. ej. la persona puede sentirse cómoda con un nivel diferente de contacto visual, o puede estar acostumbrada a contar con un espacio personal más amplio. Debes tener en cuenta que puede tener requisitos especiales con respecto a la búsqueda de ayuda para problemas de salud mental debido a su origen cultural o creencias religiosas. También puede encontrarse afectada por el impacto económico que la depresión puede generar, y eso repercutir negativamente sobre su estado y sobre su disposición a buscar ayuda.

En el caso en que la persona pertenezca a un grupo minoritario y/o a los grupos LGBTI, es importante que estés familiarizado con el tema y sepas de redes locales profesionales que puedan ayudarla. Si percibes que puede haber diferencias culturales que pudieran condicionar la consulta en el equipo de salud o que estuvieran interfiriendo en tu capacidad para ayudar, deberías averiguar si hay equipos de salud en tu zona entrenados

6. Búsqueda de ayuda

Es fundamental que seas capaz de reconocer cuándo alentar a la persona a buscar ayuda profesional. Tratar la depresión tempranamente es importante para obtener los mejores resultados, por lo que no debes asumir que la depresión desaparecerá por sí misma.

Si la persona no sabe dónde obtener ayuda, debes analizar las opciones que tiene y alentarla a usar estas opciones, así como ofrecerle ayuda para buscar ayuda. Para esto, debes saber qué servicios están disponibles en tu área local, y conocer las vías locales para obtener ayuda profesional (p. ej. si para ver a un especialista hace falta tener una derivación de un médico generalista o de cabecera).

Es importante que hables sobre la búsqueda de ayuda profesional como algo "normal" (p. ej. habla de ello como una acción natural a tomar, explica que los problemas de salud mental son comunes y tratables). En este sentido, debes remarcar:

- Los beneficios de buscar ayuda profesional
- Recursos de ayuda disponibles localmente

Evita etiquetar los comportamientos o sentimientos de la persona como síntomas de depresión al hablar sobre la búsqueda de ayuda con ella. Teniendo presente que una persona con depresión necesita tomar sus propias decisiones tanto como sea posible, si acompañas a la persona a la cita no debes hacerte cargo por completo.

Debes saber que la depresión a menudo no es reconocida por los profesionales de la salud y que puede tomar algún tiempo para obtener un diagnóstico. Si este es el caso, debes alentar a la persona a no darse por vencida. Si estuvo en tratamiento previamente y le resultó de utilidad, puedes sugerir volver a consultar al mismo profesional o centro asistencial si sigue estando disponible.

La capacidad y el deseo de usar estrategias de autoayuda dependerán de su interés y de la gravedad de su depresión, por lo que no deberías ser demasiado enérgico o vehemente cuando se trata de alentar a usar estrategias de autoayuda (incluso aquellas que se consideran basadas en la evidencia). Debes desalentar a la persona de automedicarse, usar alcohol u otras drogas para sentirse mejor. En cambio, si está interesada en estrategias de autoayuda, deberías alentar a usar estrategias apropiadas.

7. Qué hacer si la persona no quiere ayuda

Si la persona se niega a buscar o aceptar ayuda profesional, no debes usar el engaño, la coerción o las amenazas para garantizar que reciba ayuda. Debes averiguar si hay razones específicas por las cuales no desea buscar ayuda profesional (p. ej., preocupaciones sobre cómo pagarla, no tener un médico que le gusta o preocuparse de que sea enviada al hospital), ya que algunas veces las razones se basan en creencias erróneas o se pueden superar con ayuda. Por esto, debes conocer las barreras para buscar tratamiento para la depresión, (p. ej., la persona puede sentir que nadie va a poder ayudarla realmente).

No debes empujar a la persona a buscar ayuda profesional antes de que esté lista, a menos que exista un riesgo específico de daño a sí misma o a los demás, porque si tratas de forzar o presionar a la persona para buscar ayuda, puede dejar de buscar ayuda por completo. Asimismo, es importante que le informes que, si cambia de opinión en el futuro en cuanto a buscar ayuda, puede contactarte.

8. Preocupaciones por la seguridad

Debes respetar el derecho de la persona a no buscar ayuda, a menos que creas que está en riesgo de hacerse daño a sí misma o a otros. Para estos casos, es importante que conozcas las instrucciones de primeros auxilios de salud mental sobre cómo ayudar a alguien con pensamientos o comportamientos suicidas. Debes buscar ayuda profesional inmediata si la persona experimenta alucinaciones o delirios. Si corre el riesgo de hacerse daño a sí misma o a los demás, deberías considerar tu propia seguridad y la de los demás, y tomar las medidas de protección necesarias (p.ej. contactar a la policía o al equipo de crisis de salud mental).

CÓMO BRINDAR AYUDA A UNA PERSONA CON DEPRESIÓN:

RECOMENDACIONES DE PRIMERA AYUDA EN SALUD MENTAL

Propósito de esta guía

Esta guía de primeros auxilios en salud mental para personas con problemas de depresión destinada a miembros de la comunidad sin ningún tipo de experiencia o estudio previo referido a la salud /salud mental o depresión.

El rol de la persona capacitada por medio de esta guía es poder ofrecer una ayuda inicial a la persona adulta con problemas de depresión hasta cuando ésta reciba la ayuda profesional en el caso de ser necesaria.

¿Cómo se desarrolló esta guía?

El desarrollo de esta guía fue realizado en base a la opinión de expertas y expertos de la Argentina y de Chile, tanto en cuanto a su formación profesional (médicos generalistas, psiquiatras, psicólogos, psicólogos sociales, técnicos de rehabilitación, terapeutas ocupacionales, enfermos, etc.) como personas con experiencia vivida de recuperación de depresión o como personas que han provisto apoyo personal a personas con problemas de depresión.

El equipo de Argentina y de Chile utilizó las guías de primeros auxilios existentes en Australia y realizó la adaptación correspondiente con el asesoramiento de expertos locales. Los detalles de la metodología empleada pueden consultarse en la publicación Encina E, Agrest M, Tapia-Muñoz T, Wright J, Ardila-Gómez S, Alvarado R, Leiderman E, Reavley N. Development of mental health first aid guidelines for depression: a Delphi expert consensus study in Argentina and Chile. (en proceso de publicación).

Cómo utilizar estas recomendaciones

Esta guía reúne un conjunto de orientaciones generales. Cada persona es única y es importante adaptar el apoyo que se le entregue a sus necesidades. En este sentido, puede que haya personas que no sean ayudadas aún siguiendo estas recomendaciones o que estas no sean apropiadas para algunas personas, aun cuando están formuladas en consideración a aspectos culturales de la población chilena y argentina.

Además, debe considerarse muy especialmente que algunas personas presentan junto con el problema de depresión algún otro tipo de trastornos o enfermedades que pueden requerir abordajes específicos y especializados.

Estas guías fueron desarrolladas por la Universidad de Palermo, la Universidad de Chile y la Universidad de Melbourne y están protegidas por el derecho de autor. Sin embargo, se pueden usar libremente si no es con fines de lucro.

Se recomienda citar como: Primeros Auxilios en Salud Mental, América Latina. Brindando ayuda a una persona con problemas de depresión: Guía de primeros auxilios en salud mental. Universidad de Chile, Universidad de Palermo y Universidad de Melbourne, 2021.

Esta guía se ha elaborado como parte de un conjunto de directrices sobre la mejor manera de asistir a una persona con problemas de salud mental y está disponible en *https://mhfainternational.org/current-mhfa-research/.* El conjunto de recomendaciones de primeros auxilios en salud mental en Inglés puede consultarse y descargarse en *mhfa.com.au/resources/mental-health-first-aid-guidelines*

Si tiene dudas respecto de su utilización puede dirigirse a:

Esteban Encina, esteban2@uchile.cl
Martín Agrest, magrest66@gmail.com
Thamara Tapia, thammytapia@gmail.com
Nicola Reavley, nreavley@unimelb.edu.au. +61 3 9035 7628
