# Supplementary material for: Development of mental health first-aid guidelines for depression: a Delphi expert consensus study in Argentina and Chile
Source: BMC Psychiatry. 2023 Mar 14;23:161. doi: 10.1186/s12888-023-04661-8 (PMC10013290; doi:10.1186/s12888-023-04661-8)
Supplement: Supplementary file 3 — Additional file 3: Supplementary file 3. Differences between the English-language mental health first aid guidelines for depression and those for Argentina and Chile. [file 12888_2023_4661_MOESM3_ESM.docx]

# Supplementary file 3. Differences between the English-language mental health first aid guidelines for depression and those for Argentina and Chile

|  | | |
| --- | --- | --- |
| **Changes (n)** | **Statements in English** | **Statements in Spanish** |
|  | **Section 1: How do I know if someone is experiencing Depression/Learning about depression** | **Sección 1: ¿Cómo sé si alguien está experimentando una depresión? / Aprendiendo sobre la depresión** |
| **Added (2)** | The first aider must know that there are stressors that can make the symptoms chronic, such as: other medical health problems, economic problems and family problems. | El asistente debe saber que hay factores estresantes que pueden hacer crónicos los síntomas como: otros problemas médicos de salud, problemas económicos y problemas familiares. |
|  | The first aider must know the characteristics of minority, excluded or vulnerable groups, women, children and the elderly, who may present particular characteristics or symptoms of depressive disorders. | El asistente debe conocer las características de los grupos minoritarios, excluidos o vulnerables, mujeres, niños y personas mayores, que pueden presentar características o síntomas particulares de los trastornos depresivos. |
| **Excluded (1)** | The first aider should learn more about depression by: - Seeking advice from people who have experienced and recovered from depression | El asistente debe aprender más acerca de la depresión por medio de: - Buscar consejos de personas que han experimentado y se han recuperado de depresión |
|  |  |  |
|  | **Section 2: How should I approach someone who may be experiencing depression** | **Sección 2: ¿Cómo debo acercarme a alguien que puede estar experimentando depresión?** |
| **Added (5)** | The first aider must know that delaying the approach or not taking advantage of an opportunity to identify a depression problem early can lead the person to have more problems in the future. | El asistente debe saber que demorar el acercamiento o no aprovechar una oportunidad para identificar tempranamente un problema de depresión pueden llevar a la persona a tener mayor problemas en el futuro. |
|  | The first aider must be aware of the importance of personal meetings to help the person and of the limitations of messages or other forms of delayed contact (for example, WhatsApp messages). | El asistente debe estar advertido de la importancia de los encuentros personales para ayudar a la persona y de las limitaciones de mensajes u otras formas de contacto diferido (por ejemplo, los mensajes de Whatsapp). |
|  | The first aider must evaluate the relevance of using digital platforms to communicate with the person when meetings in person are not possible. | El asistente debe evaluar la pertinencia de utilizar plataformas digitales para comunicarse con la persona cuando los encuentros en persona no sean posibles. |
|  | The first aider should be aware that a person may feel exhausted or invaded if the first aider asks too insistently about how they are feeling. | El asistente debe ser consciente de que una persona puede sentirse agotada o invadida si el asistente le pregunta demasiado insistentemente acerca de cómo se siente. |
|  | The first aider must explore the sociocultural context of the person before suggesting actions that may not be feasible. | El asistente debe explorar el contexto sociocultural de la personas antes de sugerir acciones que puedan no ser viables. |
| **Excluded (6)** | If the first aider thinks someone is depressed, she should try to spend time with the person and gently propose her concerns (eg, mention that she seems depressed today). | Si el asistente de primeros auxilios piensa que alguien está deprimido, debería tratar de pasar tiempo con la persona y plantearle sus preocupaciones con delicadeza (p.ej., mencione que tiene la impresión de verla deprimida hoy). |
|  | The first aider should be open to any opportunity to discuss her concerns with the person. | El asistente debe estar abierto a cualquier oportunidad de hablar sobre sus preocupaciones con la persona. |
|  | If the first aider is concerned about someone who may be depressed, they should: -Allow the person to choose when to open up and talk about what is happening. | Si el asistente está preocupado por alguien que puede estar deprimido, debería: - Permitir que la persona elija cuándo abrirse y hablar de lo que le pasa. |
|  | The first aider must respect the person's interpretation of the signs and symptoms. | El asistente debe respetar la interpretación de la persona respecto de los signos y síntomas. |
|  | The first aider must be aware that the person's thoughts, feelings and beliefs represent their own reality and the first aider must be prepared to accept them without question. | El asistente debe ser consciente de que los pensamientos, sentimientos y creencias de la persona representan su propia realidad y el asistente debe estar preparado para aceptarlos sin cuestionarlos. |
|  | The first aider should consider that the person might already have information and know about depression. | El asistente debe considerar que la persona podría ya tener información y saber sobre la depresión. |
|  |  |  |
|  | **Section 3: How can I be supportive** | **Sección 3: ¿Cómo puedo dar apoyo?** |
| **Added (15)** | The first aider must respect the autonomy of the person unless they takes the risk of harming themselves or third parties and are not in a position to decide for themselves. | El asistente debe respetar la autonomía de la persona salvo que corra riesgos de hacerse daño o hacerles daño a terceros, y no esté en condiciones de decidir por sí misma. |
|  | Prior acceptance of the person, the first aider should try to involve significant others (such as relatives, relatives, or friends), unless the situation is so serious that the first aider should consider involving others even if they do not have the acceptance of the person. | Previa aceptación de la persona, el asistente debe tratar de involucrar a otros significativos (como familiares, allegados, o amigos), a menos que la situación sea de tanta gravedad que el asistente deba considerar involucrar a otros por más que no cuente con la aceptación de la persona. |
|  | The first aider must be aware that if what he says to the person is not in tune with his state of mind, it may be perceived by the person as a lack of empathy or not being understood. | El asistente debe ser consciente de que SI lo que dice a la persona no sintoniza con el estado de ánimo de ésta puede ser percibido por la persona como falta de empatía o no estar siendo comprendida. |
|  | The first aider must clearly explain that depression is not a moral or capacity issue, but rather a health issue. | El asistente debe explicitar claramente que la depresión no es un tema moral ni de capacidad, sino que es un tema de salud. |
|  | The first aider should be familiar with typical cognitive aspects of depression, for example negative thoughts, and know how to approach them when they are present. | El asistente debiese estar familiarizado con aspectos cognitivos típicos de la depresión, por ejemplo pensamiento negativos, y saber cómo aproximarse cuando estos están presentes. |
|  | The first aider, along with accompanying them to seek professional help, can suggest activities to the person and avoid isolating themselves, even if they do not enjoy them at first. | El asistente, junto con acompañar a buscar ayuda profesional, puede sugerir a la persona realizar actividades y evitar aislarse, aunque al principio no las disfrute. |
|  | The first aider must know that, especially when the person is feeling bad, it is positive to reinforce all those attitudes or actions that show improvement or are an achievement. | El asistente debe saber que, especialmente cuando la persona se está sintiendo mal, es positivo reforzar todas aquellas actitudes o acciones que muestren una mejoría o sean un logro. |
|  | The first aider must evaluate in each case if they is suitable to provide first aid to that person. | El asistente debe evaluar en cada caso si es idóneo para prestar primeros auxilios a esa persona. |
|  | The first aider must learn to know theirs limits and ask for help when they have exceeded them. | El asistente debe aprender a conocer sus límites y pedir ayuda cuando los ha excedido. |
|  | The first aider must be clear that when it is time to stop accompanying the person, they must have prepared them first. | El asistente debe tener claro que llegado el momento de dejar de acompañar a la persona debe haberla preparado primero. |
|  | The first aider must keep in mind that it is important to transmit warmth, security and tranquility to the person. | El asistente debe tener presente que es importante transmitir calidez, seguridad y tranquilidad a la persona. |
|  | The first aider should consider conveying to the person that the recovery process is gradual, and that sometimes there are setbacks before moving forward. | El asistente debe considerar transmitir a la persona la idea de que el proceso de recuperación es gradual, y que a veces hay retrocesos antes de seguir avanzando. |
|  | The first aider should not be restricted to talking only about aspects related to depression if the person is willing to talk about other topics. | El asistente no debiese restringirse a hablar únicamente de aspectos relacionados con la depresión si es que la persona está dispuesta a hablar de otros temas. |
|  | In the event that the person is an adolescent, it is important that the first aider is trained in the specific problem (as well as in the interaction with mothers, fathers or other caregivers) and with basic knowledge of the legal regulations in force in their area. . | En el caso que la persona sea adolescente, es importante que el asistente esté entrenado en la problemática específica (así como en la interacción con madres, padres u otros/as cuidadores/as) y con conocimientos básicos de las normativas legales vigentes en su zona. |
|  | The first aider should consider the importance of being part of a network and having a personal support network in order to better accompany people with depression. | El asistente debería considerar la importancia de formar parte de una red y contar con una red de apoyo personal para poder acompañar mejor a personas con depresión. |
| **Excluded (10)** | If the person judges himself too harshly, the first aider should remind them of theirs virtues or strengths. | Si la persona se juzga a sí misma con demasiada dureza, el asistente debe recordarle sus virtudes o puntos fuertes. |
|  | If the person says that they feels that they is a weak person or a failure, the first aider should let the person know that: -They do not believe that the person is weak or that it is to have a failure | Si la persona dice que siente que es una persona débil o un fracaso, el asistente debe hacerle saber a la persona que: - No cree que la persona sea débil o que eso sea tener una falla |
|  | If the person says that they feels that they is a weak person or a failure, the first aider should let the person know that: - They do not think that he is worth less as a person. | Si la persona dice que siente que es una persona débil o un fracaso, el asistente debe hacerle saber a la persona que: - No piensa que valga menos como persona. |
|  | The first aider should ask the person if what he is doing is helpful, and what else he could do to help. | El asistente debería preguntarle a la persona si lo que está haciendo es útil, y qué otra cosa podría hacer para ayudar. |
|  | The first aider should consider telling the person that: - They is important to the first aider and their life is also important | El asistente debería considerar decirle a la persona que: - Ella es importante para el asistente y que su vida también es importante |
|  | The first aider should not adopt an overly involved or overprotective attitude towards the person. | El asistente no debe adoptar una actitud excesivamente involucrada o sobreprotectora hacia la persona. |
|  | The first aider should not speak to the person in a patronizing tone of voice and should not adopt overly sympathetic or worried looks | El asistente no debe hablarle a la persona con un tono de voz paternalista y no debe adoptar miradas excesivamente compasivas ni de preocupación |
|  | The first aider should not insist that the person do what they would normally do. | El asistente no debería insistir a la persona para que haga lo que normalmente haría. |
|  | The first aider should know that recovery, for the most part, should be person-led. | El asistente debe saber que la recuperación, en su mayor parte, debe ser dirigida por la persona. |
|  | The first aider should offer hope for a more positive future in some way that the person, even when depressed, can accept. | El asistente debería ofrecer la esperanza de un futuro más positivo en alguna forma que la persona aun estando deprimida pueda aceptar. |
|  |  |  |
|  | **Section 4: Communicating effectively** | **Sección 4: Comunicación efectiva** |
| **Added (4)** | If the first aider belongs to the person's circle, they should avoid saying phrases like "you didn't want to listen", "I told you so" or, "you don't listen to me". | Si el asistente pertenece al círculo de la persona debe evitar decir frases como “no quisiste escuchar”, “te lo dije” o, “no me haces caso”. |
|  | The first aider should try to get to know and understand the person in order to know how to best relate to them. | El asistente debe intentar conocer y comprender a la persona para saber cómo relacionarse mejor con ella. |
|  | The first aider must consider that there are people who need to be accompanied in silence, and that sometimes this allows the person to express themselves spontaneously. | El asistente debe considerar que hay personas que necesitan que las acompañen en silencio, y que a veces eso le permite a la persona expresarse espontáneamente. |
|  | The first aider should consider the possibility and be prepared in case the person: 1. Reacts annoyed, aggressively, or don't take positively to something you say. 2. Feels embarrassed to talk about their problems and adopts an avoidant attitude. | El asistente debe considerar la posibilidad y estar preparado por si la persona: 1. reacciona con molestia, agresivamente, o no toma positivamente algo que le diga. 2. Siente vergüenza de hablar de sus problemas y adopta una actitud evitativa. |
| **Excluded (5)** | The first aider should ask the person if stress is a problem for them and, if it is, encourage them to find ways to reduce stress in their life. | El asistente de primeros auxilios debería preguntarle a la persona si el estrés es un problema para ella y, si lo es, alentarla a buscar formas de reducir el estrés en su vida. |
|  | The first aider should encourage the person to talk about their thoughts, feelings, symptoms, and any other problems they are experiencing. | El asistente debe alentar a la persona a hablar sobre sus pensamientos, sentimientos, síntomas y cualquier otro problema que esté experimentando. |
|  | The first aider should explore with the person how their symptoms affect their daily life. | El asistente debe explorar con la persona cómo sus síntomas afectan su vida diaria. |
|  | If the person finds it difficult to talk openly about their thoughts and feelings with the first aider, the first aider should tell them about available services where they can talk to another person, eg a telephone counseling service. | Si a la persona le resulta difícil hablar abiertamente sobre sus pensamientos y sentimientos con el asistente, el asistente debe informarle acerca de los servicios disponibles donde pueden hablar con otra persona, p.ej. un servicio de asesoramiento telefónico. |
|  | The first aider should use the following verbal skills to show that they are listening: - Use minimal expressions when necessary to keep the conversation going with the person (such as "I understand", "Mmmm" or another way to help keep the person the conversation) (90.4) | El asistente debe usar las siguientes habilidades verbales para mostrar que está escuchando: - Usar expresiones mínimas cuando sea necesario para mantener la conversación con la persona (como p.ej., "entiendo", "Mmmm" u otra manera de ayudar a que siga la conversación) (90.4) |
|  |  |  |
|  | **Section 5: Difficulties the first aider may encounter** | **Sección 5: Dificultades que el asistente podría encontrar** |
|  | If the person gets upset during the conversation, the first aider should: 4. Consider asking what made them upset. | Si la persona se enoja durante la conversación, el asistente debería: 4. Considerar preguntar qué fue lo que le causó molestia. |
| **Added (7)** | If the person gets upset during the conversation, the first aider should: 5. Try to politely redirect the conversation and reiterate their willingness to help. | Si la persona se enoja durante la conversación, el asistente debería: 5. Intentar reencauzar el diálogo amablemente y reiterar su disposición a ayudar. |
|  | If the person gets upset during the conversation, the first aider should: 6. Consider taking a break in the conversation and ask the person how they want to continue. | Si la persona se enoja durante la conversación, el asistente debería: 6. Considerar tomar un descanso en la conversación y preguntar a la persona cómo quiere seguir. |
|  | If the person gets angry during the conversation, the first aider should: 7. In the case of not being able to control the situation, try to reassure the person and consider seeking support. | Si la persona se enoja durante la conversación, el asistente debería: 7. En el caso de no poder controlar la situación, intentar tranquilizar a la persona y considerar buscar apoyo. |
|  | The first aider must take into account that the person may be affected by the economic impact that depression can generate, and this may have a negative impact on their condition and their willingness to seek help. | El asistente debe tener en cuenta que la persona puede estar afectada por el impacto económico que la depresión puede generar, y eso repercutir negativamente sobre su estado y sobre su disposición a buscar ayuda. |
|  | In the event that the person belongs to a minority group and/or LGBTI groups, it is important that the first aider is familiar with the subject and knows of local professional networks that can help the person. | En el caso en que la persona pertenezca a un grupo minoritario y/o a los grupos LGBTI es importante que el asistente esté familiarizado con el tema y sepa de redes locales profesionales que puedan ayudar a la persona. |
|  | If the first aider perceives that there may be cultural differences that could condition the consultation in the health team or that are interfering with their ability to help the person, the first aider should find out if there are health teams in their area trained in issues of cultural differences in order to better guide the person. | Si el asistente percibe que puede haber diferencias culturales que pudieran condicionar la consulta en el equipo de salud o que estuvieran interfiriendo en su capacidad para ayudar a la persona, el asistente debería averiguar si hay equipos de salud en su zona entrenados en temas de diferencias culturales para poder orientar mejor a la persona. |
| **Excluded (3)** | The first aider should use the following nonverbal skills to reinforce their nonjudgmental communication: - Sit next to the person and at an angle to them, rather than directly in front of them (98.1) | El asistente debe usar las siguientes habilidades no verbales para reforzar su comunicación sin prejuicios: - Siéntese junto a la persona y en ángulo hacia ella, en lugar de directamente enfrente de ella (98.1) |
|  | If there were cultural differences that were interfering with the caregiver's ability to help the person, it would be appropriate to suggest that the person refer to a mental health service that is sensitive to cultural differences. | Si hubiera diferencias culturales que estuvieran interfiriendo en la capacidad del asistente de ayudar a la persona, sería indicado sugerir que la persona se dirija a un servicio de salud mental que sea sensible a las diferencias culturales. |
|  | If cultural differences are interfering with the first aider's ability to help the person, you should openly discuss with the person what would be culturally appropriate and how it might be possible for them to receive help or consider talking to someone culturally close to the person about it. | Si las diferencias culturales están interfiriendo con la capacidad del asistente para ayudar a la persona, debe conversar abiertamente con la persona qué sería lo culturalmente apropiado y cómo sería posible para ella recibir ayuda, o considerar consultar al respecto con alguien culturalmente cercano a la persona. |
|  |  |  |
|  | **Section 6: Help-seeking** | **Sección 6: Búsqueda de ayuda** |
| **Added (2)** | The first aider should discourage the person from self-medicating. | El asistente debe desalentar a la persona de automedicarse. |
|  | If the person was in treatment before and found it helpful, the first aider can suggest going back to the same professional or health center if it is still available. | Si la persona estuvo en tratamiento previamente y le resultó de utilidad, el asistente puede sugerir volver a consultar al mismo profesional o centro asistencial si sigue estando disponible. |
| **Excluded (7)** | The first aider should ask the person if they think they would benefit from professional help. | El asistente debe preguntarle a la persona si cree que se beneficiaría de la ayuda profesional. |
|  | The first aider should have some general knowledge about the types of treatment that can be helpful for depression. | El asistente debe tener algunos conocimientos generales sobre los tipos de tratamiento que pueden ser útiles para la depresión. |
|  | The first aider should encourage the person to make a list of questions that they should discuss with the health professional at their first appointment. | El asistente debe alentar a la persona a hacer una lista de preguntas que debe discutir con el profesional de la salud en su primera cita. |
|  | Before suggesting self-help strategies, the first aider should ask the person what strategies she is currently using or which she has used in the past that she has found helpful. | Antes de sugerir estrategias de autoayuda, el asistente debería preguntarle a la persona qué estrategias está utilizando actualmente o cuáles de las que ha utilizado en el pasado le resultaron útiles. |
|  | The first aider should encourage the person to use self-help strategies that have helped the person in the past. | El asistente debe alentar a la persona a usar estrategias de autoayuda que hayan ayudado a la persona en el pasado. |
|  | If the person is interested in self-help strategies, the first aider should: - Encourage them to consult reliable sources about what is likely to be most useful (eg a Ministry of Health website) | Si la persona está interesada en estrategias de autoayuda, el asistente debería: - Alentarla a consultar fuentes confiables sobre lo que probablemente sea más útil (p.ej. un sitio web dependiente del Ministerio de Salud) |
|  | If the person is interested in self-help strategies, the first aider should: - Discuss with them a variety of self-help strategies that might be helpful | Si la persona está interesada en estrategias de autoayuda, el asistente debería: - Discutir con ella una variedad de estrategias de autoayuda que podrían ser útiles |
|  |  |  |
|  | **Section 7: What to do if the person doesn’t want help** | **Sección 7: Qué hacer si la persona no quiere ayuda** |
| **Added (0)** | None | Ninguno |
| **Excluded (1)** | If, despite the first aider's efforts, the person is still unwilling to seek or accept help, the first aider must respect their wishes, unless there is a risk of harming themselves or another person. | Si, a pesar de los esfuerzos del asistente, la persona aún no está dispuesta a buscar o aceptar ayuda, el asistente debe respetar sus deseos, a menos que exista el riesgo de dañarse o a de dañar a otra persona. |
|  |  |  |
|  | **Section 8: Concerns for safety** | **Sección 8: Preocupaciones por la seguridad** |
| **Added (0)** | None | Ninguno |
| **Excluded (2)** | If the person is at risk of harm to themselves or others, the first aider should: - Involve the person in decisions about who else should be told about the risk of harm | Si la persona corre el riesgo de hacerse daño a sí misma o a los demás, el asistente debería: - Involucrar a la persona en las decisiones sobre a quién más se le debe informar sobre el riesgo de daño |
|  | If the person is at risk of harming themselves or others, the first aider should: - Ask the person to take steps to get help, e.g. see a GP | Si la persona corre el riesgo de hacerse daño a sí misma o a los demás, el asistente debería: - Pedirle a la persona que tome medidas para obtener ayuda, p.ej. ver a un médico de cabecera |
